# Cellular Therapies in Pediatric Liver Diseases

**DOI:** 10.3390/cells11162483

**Published:** 2022-08-10

**Authors:** Sunitha Vimalesvaran, Jessica Nulty, Anil Dhawan

**Affiliations:** Pediatric Liver GI and Nutrition Center and Mowat Labs, Institute of Liver Studies, King’s College London and King’s College Hospital, London SE5 9RS, UK

**Keywords:** pediatric liver transplantation, hepatocyte transplantation, mesenchymal stromal cell, acute liver failure

## Abstract

Liver transplantation is the gold standard for the treatment of pediatric end-stage liver disease and liver based metabolic disorders. Although liver transplant is successful, its wider application is limited by shortage of donor organs, surgical complications, need for life long immunosuppressive medication and its associated complications. Cellular therapies such as hepatocytes and mesenchymal stromal cells (MSCs) are currently emerging as an attractive alternative to liver transplantation. The aim of this review is to present the existing world experience in hepatocyte and MSC transplantation and the potential for future effective applications of these modalities of treatment.

## 1. Introduction

Liver transplantation remains the accepted treatment for end-stage liver disease and many liver-based metabolic disorders. However, there remain constraints of scarcity of donor livers, high costs involved in transplantation, risks of complications post-operatively and the need for lifelong immunosuppression. The impact of liver transplantation on quality of life cannot be underestimated with a recent study suggesting that only 26% of a pediatric cohort achieved “meaningful survival” 20-years post-transplantation [1]. The advent of auxiliary liver transplantation, where a partial liver lobe is transplanted whilst leaving the native liver in situ, has shown that whole liver replacement is not necessary for the restoration of liver function in children with acute liver failure. Up to 70% of these patients can be weaned off immunosuppression due to spontaneous native liver regeneration. [2]. Hence alternative therapies like cellular therapies, gene therapies and use of small molecules are under constant review as alternatives to whole liver replacement.

Human primary hepatocyte transplantation (HTx) is the primary form of cell therapy in paediatric liver disease, having been studied extensively in animal models of human liver disease with several clinical series of human hepatocyte transplantation with encouraging results, for over 30 years. It is however, limited by the shortage of donor tissues, from which hepatocytes of good quality can be isolated, as well as the impact of rejection against allogenic cell graft in the longer term and the need for immunosuppression when used for the treatment of liver based metabolic disorders.

As such, other modalities of cell therapy are being considered, predominantly focusing on the therapeutic potential of stromal cells. Their use is particularly appealing due to the fact that the cells are readily available and have the potential to expand in vitro and in vivo. Some cells can also be isolated from the recipient itself with auto-transplantation avoiding the need for immunosuppression.

The aim of this review is to summarize the current state of the art for cell therapies in pediatric liver disease. We will highlight the advantages and disadvantages of each cellular modality, explore its present use in clinical trials and how potential limitations can be addressed.

Our review is restricted to primary human hepatocytes and MSC, which has the most current evidence in the pediatric population and not stem cell generated hepatocytes.

## 2. Hepatocyte Transplantation (HTx)

### 2.1. Biology and Origins

The hepatocyte is the functional unit of the liver, performing synthetic and detoxification functions. HTx has emerged as an alternative treatment modality to liver transplantation, providing missing hepatic function to patients once engrafted. Hepatocytes are generally isolated from donor livers unsuitable for transplantation due to prolonged warm or cold ischaemia times, steatosis, anatomic disparities and lately from neonatal donors. Cells are isolated under good manufacturing practice (GMP) using a three-step collagenase perfusion technique, which has been previously described [3]. Thereafter, hepatocytes are separated from other non-parenchymal cells using low speed centrifugation. Isolated hepatocytes can be effectively used either fresh or cryopreserved for later utilization

There are many advantages of HTx over orthotopic liver transplantation. It is less invasive and more economical, without the need for complex surgery. It also has the advantage of keeping the native liver in situ, allowing for potential future option of gene therapy or use of small molecule-based therapies. HTx can also be repeated if necessary, and has the advantage of being readily available, particularly when using cryopreserved cells. Physiological benefit is postulated when 5–10% of the liver mass is replaced, and thus multiple patients can benefit from one donor liver, saving other organs for patients who may benefit only from liver transplantation [4].

In 1977, Groth et al. demonstrated a significant decrease in hyperbilirubinaemia in a rat model for Crigler-Najjar syndrome Type I, 28 days after HTx [5]. A number of pre-clinical trials later, led to the first in human study of HTx in 1992. 10 patients with liver cirrhosis received autologous hepatocytes, but with no clear benefit [6].

### 2.2. HTx in Paediatric Liver Disease

Multiple clinical series have since been reported in patients with liver disease, with the most significant effects seen in patients with metabolic disorders (Table 1).

#### 2.2.1. HTx for the Treatment of Metabolic Disorders

Crigler-Najjar syndrome, phenylketonuria and factor VII deficiency are all liver based single gene defects, which lead to a reduced or absent function of the encoded gene product. It has been estimated that a cell engraftment corresponding to ~ 5–10% of liver mass is sufficient to overcome the gene defect and improve clinical outcome [7]. Although liver transplantation is the only curative therapy for these diseases, HTx has a promising treatment potential by replacing the diseased hepatocytes and restoring necessary function. In 1998, the first sustained effect of HTx for a single gene defect was demonstrated in a 10-year-old girl with Crigler-Najjar syndrome Type I who was on the liver transplantation waiting list. After a dose of 7.5 × 10^9^ hepatocytes, partial correction of hyperbilirubinemia was achieved and sustained for 11 months [8]. Since then, HTx has been used to treat numerous other genetic liver diseases with varying success (Table 1).

In our experience at the King’s College Hospital, London, similar to others, HTx for the treatment of liver-based metabolic defects has resulted in varying successes. We have shown promising success in using HTx for the treatment of Crigler-Najjar syndrome [9] ornithine transcarbamylase (OTC) deficiency [10], and inherited factor VII deficiency [9]. Although our HTx metabolic program is suitable as a bridge to transplantation, new approaches which can improve hepatocyte engraftment and sustained functionality are needed and are under development before HTx can become a standard of care for liver-based metabolic diseases.

#### 2.2.2. HTx for Treatment of Acute Liver Failure (ALF)

HTx has also shown some promising clinical success in the treatment of acute liver failure (ALF). ALF is a rapid decline in liver function over the course of days or weeks. Once ALF progresses in severity, the only medical treatment available is orthotopic liver transplantation, without which survival rates are very low [11]. HTx may be an effective treatment option whilst the patient waits for a donor liver to become available. HTx has been used to treat various cases of ALFs which have occurred following Dilantin [12], halothane [13] and multiple polysubstance misuse [12] showing promising improvements in encephalopathy and ammonia concentrations (Table 2). When human foetal hepatocytes were used to treat ALF, the overall survival of the treated group was 43% compared to 33% in the matched controls [14].

Although utilizing HTx for the treatment of ALF appears promising, it requires hepatocytes to be transplanted into an extremely harsh environment containing high levels of cellular necrosis and apoptosis [15]. As such, novel approaches are being examined to overcome this harsh microenvironment including implanting cells in alternative sites such as the peritoneum [16] and lymph nodes [17].

Another strategy to protect transplanted hepatocytes from the hostile microenvironment of a failing liver is to encapsulate the implanted cells within a biocompatible polymer. We have developed a technique using alginate to form hepatocyte microbeads [18] which can be infused into the peritoneal cavity of the patient, to temporarily replace the failing liver until regeneration can occur. These microbeads have been shown to be safe and, importantly, demonstrated promising efficacy in a pediatric cohort with ALF [19]. Since then, we have refined our hepatocyte microbead prototype. Progressing to a microbead which now involves multiple cell types and an improved hydrogel that better supports the cell function in vitro as well as in vivo, in preclinical studies (unpublished). Furthermore, a recent study has shown these types of alginate microbeads containing hepatocytes can be cryopreserved with some maintenance of hepatic functions once thawed, indicating the possibility of an off-the-shelf product being available for rapid treatment of ALFs [20]. These improved beads are currently being tested in Phase I/II clinical trial to show safety and efficacy (EudraCT Number: 2019-003916-29).

**Table 1:** Hepatocyte transplantation: clinical studies in patients with inborn errors of metabolism.

Reference	Liver Disease	Patients	Cell Type	Route	Dose	Outcome
(Fox et al., 1998) [8]	Crigler-Najjar syndrome type 1	1 female child (10 years)	Fresh primary hepatocytes (5-year-old donor)	Portal Vein	7.5 × 10^7^	OLT after 4 years
(Darwish et al., 2004) [21]		1 female child (8 years)	Both fresh and cryopreserved primary hepatocytes	Portal Vein	7.5 × 10^7^ (9 injections over 5 months)	OLT after 20 months
(Ambrosino et al., 2005) [22]		1 male child (9 years)	Fresh primary hepatocytes (47-year-old donor)	Portal Vein	7.5 × 10^7^	OLT after 5 months
(Dhawan et al., 2006) [23]		1 male child (18 months); 1 female child (3 years)	Cryopreserved primary hepatocytes	Portal Vein	4.3 × 10^7^ 2.1 × 10^7^	OLT after 8 months
(Allen et al., 2008) [24]		1 female child (8 years)	Fresh primary hepatocytes (7-year-old donor)	Portal Vein	1.4 × 10^7^	OLT after 11 months
(Lysy et al., 2008) [25]		1 female child (9 year); 1 female child (1 year)	Both fresh and cryopreserved primary hepatocytes	Porth-a-cath in jejunal vein; Broviac in portal vein	6.1 × 10^7^ (18 infusions from 3 different donors) 2.6 × 10^7^ (14 infusions from 1 donor)	OLT after 6 months OLT after 4 months
(Grossman et al., 1995) [26]	Familial hypercholesterolemia	Five patients (7–41 years)	Fresh primary hepatocytes transduced through retrovirus-mediated gene transfer for LDLR gene	Portal Vein	1.0–3.2 × 10^7^	Variable and transient response
(Dhawan et al., 2004) [9]	Factor VII deficiency	1 child (3 months); 1 child (3 years)	Both fresh and cryopreserved primary hepatocytes	Portal Vein	1.1 × 10^7^ 2.2 × 10^7^	OLT after 7 months OLT after 8 months
(Muraca et al., 2002) [27]	Glycogen storage disease Type I	1 female adult (47 years)	Fresh primary hepatocytes	Portal Vein	2 × 10^7^	9 months after trans- plantation, patient on normal diet and can fast for 7 h without experiencing hypoglycaemia
(Lee et al., 2007) [28]		1 male adult (18 years)	Both fresh and cryopreserved primary hepatocytes	Portal Vein	2 × 10^9^ for first infusion; 1 × 10^9^ for second and 3 × 10^9^ for final infusion	250 days after HTx patient on a normal diet
(Sokal, Smets, Bourgois, Van Maldergem, et al., 2003) [29]	Infantile Refsum’s disease	1 female child (4 years)	Both fresh and cryopreserved primary hepatocytes	Portal Vein	1.1 × 10^9^ for first infusion; 1.4 × 10^8^ and 9 × 10^7^ on day 3, 1.84 × 10^8^ and 2.43 × 10^8^ on day 4, and 1.96 × 10^8^ on day 5	Continued metabolic improvement 1 year after HTx
(Dhawan et al., 2006) [23]	Progressive familial intrahepatic cholestasis Type 2	2 children (18 months and 3 years)	Fresh primary hepatocytes	Portal Vein	0.2 × 10^7^ 0.4 × 10^7^	OLT after 5 months OLT after 14 months
(S. C. Strom et al., 1997) [13]	OTC deficiency	1 male child (5 years)	Fresh primary hepatocytes	Portal Vein	1 × 10^7^	Death 42 days later
(Horslen et al., 2003) [30,31]		1 male child (10 h old)	Both fresh and cryopreserved primary hepatocytes	Portal Vein	4 × 10^9^ for first infusion; further 3.3 × 10^9^ between days of life 37 and 51; 1.7 × 10^9^ between days 113 and 116	OLT at 6 months
(Stéphenne et al., 2005) [28]		1 male child (14 months)	Cryopreserved primary hepatocytes	Portal Vein	2.4 × 10^7^ 10 infusions over 16 weeks	OLT after 6 months
(Puppi et al., 2008) [10]		1 male child (1 day)	Both fresh and cryopreserved primary hepatocytes	Portal Vein	1.74 × 10^9^; 7 infusions over the first month of life and 1 infusion at 5 months.	APOLT at 7 months
(Meyburg et al., 2009) [32]		1 male child (6 h); 1 male child (9 days)	Cryopreserved primary hepatocytes from one donor (9 days old)	Portal Vein	9.4 × 10^8^ in 3 infusions; 8.7 × 10^8^ in 2 infusions	Death at 4 months; Listed for OLT 5 months after HTx
(Stéphenne et al., 2006) [33]	ASL deficiency	1 female child (3 years)	Both fresh and cryopreserved primary hepatocytes	Portal Vein	1.7 × 10^9^ in 7 infusions over 1 month period; 2.5 months after first infusion patent received a further 10 × 10^9^ cells over 2 days; two months later a further 1× 10^9^ cells	OLT after 18 months
(Meyburg et al., 2009) [32]	CPS1 deficiency	1 male child (10 weeks)	Both fresh and cryopreserved primary hepatocytes	Portal Vein	1.87 × 10^9^ over 6 infusions	Listed for OLT 7 months after HTx
(Meyburg et al., 2009) [32]	Citrullinemia	1 female child (3 years)	Both fresh and cryopreserved primary hepatocytes	Portal Vein	1.89 × 10^9^ over 4 infusions	Protein intake could be increased 10 months after HTx

**Table 2:** Hepatocyte transplantation: clinical studies in patients with Acute Liver Failure; OLT Orthotopic liver transplantation.

Reference	Liver Disease	Patients Treated	Cell Type	Route	Dose	Outcome
(Soriano et al., 1997) [34]	Drug-induced liver failure	16 years; 12 years; 10 years	Cryopreserved primary hepatocytes	Portal Vein	4 × 10^7^–4 × 10^9^	Death on day 2; Death on day 7; Death on day 7
(Bilir et al., 2000) [35]		1 female adult (32 years); 1 male adult (35 years); 1 male adult (55 years)	Cryopreserved primary hepatocytes	Intrasplenic	1.3 × 10^9^; 1 × 10^10^; 3.9 × 10^10^	Death on day 14; Death on day 20; Death in 6 h
(Strom et al., 1999) [36]		1 female teenager (13 years); 1 female adult (43 years)	NA	Portal Vein	NA	Death on day 4; Death on day 35
(Fisher and Strom 2006) [37]		1 female adult (27 years); 26 years; 21 years; 35 years; 35 years; 51 years	NA	Intrasplenic; Intrasplenic; Intrasplenic; Portal Vein; Portal Vein; Portal Vein	2.8 × 10^7^; 3 1.2 × 10^9^; 3 infusions of 9 × 10^8^, 9 × 10^8^ and 2.5 × 10^7^; 5.4 × 10^9^; 3.7 × 10^9^; 3.9 × 10^9^	OLT on day 10; OLT on day 2; Death on day 1; Death on day 18; Full recovery; Death on day 3
(Habibullah et al., 1994) [14]		1 female adult (32 years); 1 male adult (29 years); 1 female adult (20 years); 1 female adult (20 years); 1 female adult (24 years)	Fresh foetal hepatocytes	Intraperitoneal	6 × 10^7^ kg body weight	Death in 30 h; Death in 37 h; Death in 48 h; Full recovery; Full recovery
(Fisher and Strom 2006) [37]	Viral-induced acute liver failure	4 years; 54 years	NA	Portal Vein	2 infusions of 1.7 × 10^9^; 6.6 × 10^9^	Death on day 2; Death on day 7
(Bilir et al., 2000b) [35]		1 female adult (29 years); 1 female adult (65 years)	Cryopreserved primary hepatocytes	Portal Vein and intrasplenic	1 × 10^10^; 3 × 10^10^	Death in 18 h. Death on day 52
(Strom et al., 1999) [36]		1 female adult (28 years); 1 female adult (37 years); 1 male adult (43 years)	NA	Intrasplenic; intrasplenic; Portal Vein	NA	OLT on day 3; Death on day 5; OLT on day 1
(Fisher et al., 2000b) [12]		1 female adult (37 years)	NA	intrasplenic	1.2 × 10^8^	Full recovery
(Habibullah et al., 1994b) [14]		1 female adult (40 years)	Fresh foetal hepatocytes	Intraperitoneal	6 × 10^7^/kg body weight	Death in 13 h
(Stephen C. Strom et al., 1997b) [38]		1 female adult (40 years)	Cryopreserved primary hepatocytes	Intrasplenic	7.5 × 10^6^	Death on Day 4 due to ICP monitor complications
(Soriano et al., 1997)	Idiopathic acute liver failure	3 years; 5 years	Cryopreserved primary hepatocytes	Portal Vein	4 × 10^9^	Full recovery; OLT on day 4
(Fisher and Strom 2006) [37]		3.5 months; 23 years; 48 years	NA	Portal Vein; intrasplenic; Portal Vein	1.8 × 10^8^; 2.86 × 10^8^; 7.5 × 10^8^	OLT on day 1; OLT on day 5 and death on day 13; Death on day 1
(Habibullah et al., 1994a) [14]		1 male child (8 years)	Fresh foetal hepatocytes	Intraperitoneal	60 × 10^6^/kg body weight	Full recovery
(Schneider et al., 2006) [39]	Mushroom-poisoning-induced acute liver failure	1 female (64 years)	Cryopreserved primary hepatocytes	Portal Vein	8 × 10^9^	Full recovery
(Strom et al., 1999) [36]	Postsurgical acute liver failure	1 male (69 years)	NA	Intrasplenic	NA	Death on day 2
(Khan et al., 2004) [40]	Acute liver failure induced by acute fatty liver of pregnancy	1 female (26 years)	Fresh Foetal hepatocytes	Intraperitoneal	3 × 10^8^	Full recovery
(Stephen C. Strom et al., 1997b) [38]	Alpha 1 anti-trypsin	1 female adult (52 years)	Cryopreserved primary hepatocytes	Intrasplenic	2.2 × 10^7^	OLT on Day 2
(Stephen C. Strom et al., 1997b) [38]	TPN/Sepsis	1 male child (6 months)	Cryopreserved primary hepatocytes	Intrasplenic	5.2 × 10^7^	Life support stopped on day 7

### 2.3. Future Directions of HTx

The routes of transplantation that have been used include intraportal, intraperitoneal or intrasplenic administration, with most centers using a dose of up to 2 × 10^8^ cells/kg of body weight (into the portal circulation), as the recommended upper limit per infusion, with multiple infusions being done when higher number of cells are required. Whilst the safety has been well described with good short-term outcomes, the longer term improvement of hepatic function is suboptimal due to the poor engraftment of cells and rejection, in spite of immunosuppression [41].

The issue around cell engraftment is influenced by the quality of hepatocytes that are isolated, which tends to be poor particularly when they are isolated from liver tissues that are rejected for transplantation for prolonged ischaemic time, severe fatty liver, non-heart beating and older donors. It is important to evaluate the cell quality prior to transplantation—with many centers accepting cell viability of equal or higher than 60% for clinical uses using trypan blue. Other, more specific assays to detect apoptosis and metabolic function are also increasingly being used.

Good cell engraftment is key to HTx success. In an effort to enhance hepatocyte engraftment, multiple strategies have been carried out to precondition the recipient’s liver and give a selective advantage to the transplanted cells such as portal vein embolization—a safe and well-tolerated procedure [42], partial hepatectomy—used in two patients with Crigler-Najjar syndrome Type 1 [43] and liver irradiation—a promising new preconditioning approach [44]. These techniques have been used to varying degrees of success and a better understanding of the utility of these techniques would probably improve outcomes of HTx.

Transplanted hepatocyte loss is another important consideration in the failure of HTx. Allogenic hepatocytes are immunogenic, unlike the immune-privileged liver, which allows for graft tolerance and withdrawal of immunosuppression in some cases. Both the innate and adaptive immunity play an important role in the rejection of transplanted hepatocytes. A number of immunosuppressive protocols have been utilized and adapted for HTx, mainly consisting of steroids and calcineurin inhibitors, to varying degrees of success. The instant blood mediated inflammatory response (IBMIR) is a significant phenomenon that leads to substantial cell loss when infused into the portal circulation. Alpha-one –antitrypsin use in the hepatocyte infusion has shown promise to dampen this response.

The optimized protocol for production of GMP-grade alginate encapsulated microencapsulated hepatocytes, which have a semi-permeable polymerized structure, protects the cells from host immune attack. We previously demonstrated that the ultrapure sodium alginate that is used does not elicit immune activation in vitro. This approach allows cell transplantation without using immunosuppression [18].

## 3. Mesenchymal Stromal Cells (MSC) Transplantation

### 3.1. Biology and Origins

Friedenstein et al. first isolated and identified MSCs in 1968 [45]. They are a subtype of adult fibroblast-like cells with the potential for self-renewal and a high proliferative ability. They were originally identified in the bone marrow, but because of the small number of cells that are available (0.01 to 0.001% of total bone marrow cells) [46] and the difficulty of isolating cells from the bone marrow, scientists have explored alternative sources. They have been successfully isolated from different tissue sources including synovial membrane [47], adipose tissue (AT) [48], umbilical cord (UC) blood, amniotic fluid (AF) [49] and the placenta [50]. The common feature of MSCs is the ability to differentiate into adipocyte, cartilage and osteogenic tissue. UC sources of MSCs have been of particular interest, due to the various compartments that it can be isolated from including the umbilical vein, arteries and Wharton’s jelly as well as the fact that higher quantities of more primitive MSCs that are isolated from UC tissue compared to other tissues sources. It has also been shown that MSCs isolated from UC tend to exhibit a higher proliferative capacity compared to MSCs obtained from other sources [51]. There are significant differences in the MSCs differentiation potential from different sources, despite having similar phenotypic and antigenic profiles [52,53].

### 3.2. Mechanisms of Action in Liver Disease

#### 3.2.1. Immunomodulation by MSCs

MSCs have the potential to modulate and repair injured tissue by changing toxic immune response through a range of mechanisms including direct cell–cell interactions with injured cells or remotely, through the release of paracrine factors [54]. MSCs have the added advantage of having reduced immunogenicity due to the lack of expression of Class II major histocompatibility antigens (MHC) when unprimed and the cells do not express the majority of molecules that are detectable for immune recognition such as CD80, CD86 and CD40.

##### Immuno-Modulatory Effect of MSCs on Adaptive Immunity

T-cell proliferation is inhibited in vitro by MSCs through the secretion of soluble factors or by the direct interaction with T-lymphocytes [55]. Molecules such as transforming growth factor β (TGF- β), hepatocyte growth factor, prostaglandin E2 (PGE2) [56] and indoleamine 2,3-dioxygenase [57] are secreted by MSCs with an immunomodulatory effect on T-cell activities. The secretion of these immunomodulatory molecules can differ according to the source of the MSCs [58]. The secretion of nitric oxide (NO) by MSCs also cause inhibition of the STAT5 pathways leading to the suppression of T cell proliferation [59]. The activation of T-cells is also thought to be suppressed by the release of matrix metalloproteinases (MMP) such as MMP-2 and MMP-9 [60]. MSCs also affect the generation and development of regulatory T-cells (T-regs), which can have a positive impact on the balance of immune damage during tissue injury. It is also worth noting that the inflammatory environment is particularly important in the interaction between MSCs and T-cells.

MSCs also play a role in the inhibition of B cell proliferation thereby reducing their production of immunoglobulin. In a study by Glennie et al., murine B cell proliferation was induced with CD40 and IL-4, but subsequent co-culture with MSCs significantly inhibited their proliferation [61]. There was also a consequent stimulation in immunoglobulin production after co-culture of B-cells [62]. They also tend to alter surface expression of chemokine receptors on B-cells—particularly in the expression of CXCR4, which has a role in homing and fate of MSCs [63].

Viral infections and tumor cells induce an immune response particularly involving natural killer cells [64]. The release of soluble factors such as PGE2 and TGF-β from MSCs as well as the cell–cell interactions has been shown to reduce IL-15 secretion from NK cells [55].

##### Immuno-Modulatory Effect of MSCs on Innate Immunity

Studies have suggested that MSCs trigger polarization of classical pro-inflammatory macrophages (M1) toward alterative macrophages (M2) that secrete anti-inflammatory cytokines in vivo and in vitro [65]. This polarization is secondary to the ability of MSCs to secrete soluble factors such as interleukin-10 (IL-10) and IL-1Ra, which have been shown to dampen liver injury by promoting M2 macrophage polarization [66]. Apart from this, MSCs also have the potential of increase survival of monocytes through the upregulation of CCL18, which indirectly mediates the ability of induce Treg formation by MSCs [67].

MSCs also affect dendritic cell function by blocking differentiation of antigen-presenting cells to monocytes and decreasing their expression of anti-inflammatory molecules such as IL-12, TNFα, and IFNγ, whilst also increasing the secretion of IL-10 which can induce regulatory T-cell numbers [68].

#### 3.2.2. Anti-Fibrotic Activities of MSCs

The inflammatory and fibrotic processes in the liver are closely intertwined. In liver injury, pro-fibrotic factors such as TGF-β, platelet derived growth factor (PDGF), IL-13 and IL-4, are secreted by the resident immune cells. These play a crucial role in the activation and proliferation of hepatic stellate cells (HSCs), which transform into myofibroblasts. Myofibroblasts are responsible for the production of the extracellular matrix (ECM) in the liver [69,70,71,72]. The deposition of ECM, including collagen I, collagen III and collagen IV are pivotal in causing liver fibrosis.

The anti-fibrotic effects of MSCs can be divided into the direct and indirect effects on HSCs. Indirectly, MSCs work by modulating immune cell activity to regulate the activity of HSCs. MSCs tend to migrate to the sites of injury where they are exposed to inflammatory cytokines such as IFNγ and IL-1β [73]. There, the MSCs work by secreting various soluble mediators such as NO, PGE2, IDO, IL-6, IL-10 and HLA-γ, which results in the suppression of proliferation and activation of a variety of immune cells, and induction of Treg cells [74]. MSCs suppress immune cell activity thereby reducing fibrogenic processes and reducing ECM accumulation in liver disease.

The direct anti-fibrotic effects of MSCs on HSCs involve the inhibition of ECM production potential of HSCs and the induction of apoptosis of HSCs. MSCs secrete IL-10, HGF, TGF-β3 and TNFα, which inhibit the proliferation of HSCs and decrease ECM synthesis [71]. During direct co-culture of MSCs and HSCs, the Notch pathway is activated with significant suppression of HSC proliferation and α-SMA expression. In liver fibrosis, the activated HSCs express the tissue inhibitors of metalloproteinase (TIMP)-1 and TIMP-2, whereas MSCs are known to increase the expression of MMPs. In experimental models, this is generally associated with the resolution of fibrosis [75].

#### 3.2.3. Hepatocyte-Like Differentiation of MSCs

We have discussed the potential of HTx in improving liver function and mitigating fibrosis in preclinical and clinical studies. There are several factors that can influence the hepatic differentiation of MSCs. Firstly, when MSCs are co-cultured with hepatocytes they can differentiate into hepatocyte-like cells [76]. This has been reported in rats, mice, sheep and humans [77,78]. When MSCs are treated with a combination of several growth factors, chemical compounds and cytokines (such as HGF, fibroblast growth factor 2 epidermal growth factor, oncostatin M, dexamethasone, insulin-transferrin-selenium and/or nicotinamide), there is an increase in the expression of hepatocyte markers such as albumin, α-fetoprotein, CK18, GATA4, CK19, and HNF-3β [79]. It has also been noted that hepatic stem/progenitor cells which are isolated from the adult human liver have a better potential at differentiation into hepatocytes compared to MSCs that are isolated from tissues other than the liver [79]. Future studies are still warranted improve efficacy and consistency of hepatic differentiation from MSCs.

### 3.3. MSCs in Paediatric Liver Disease

MSCs have been used in various clinical settings with varying degrees of success, including in the treatment of degenerative and immune-mediated diseases. There has been a significant increase in the number of clinical trials investigating the use of MSCs in both acute and chronic liver disease (Table 3). The question remains over whether MSCs are more effective than the conventional standard of care and which liver conditions are best suited for MSC treatment.

The majority of trials that have been published thus far have been in adults. The majority of these studies are focused on liver cirrhosis (n = 15), whilst four studies are related to liver failure and three studies are for complications after liver transplantation. The common thread for all these diseases is that they are end-stage liver diseases and the only effective treatment at this stage is liver transplantation, which is limited by the availability of organs, surgical complications, the need for immunosuppression and the high medical and surgical costs. Therefore, an effective alternative therapy such as MSC therapy is warranted in this group of patients.

Currently there are over 1000 clinical trials (1586 trials in adults and 322 trials in the paediatric population) that are associated with MSC cell therapies that are registered with www.clinicaltrials.gov (accessed on 15 June 2022). Additionally, MSC cell therapy has been employed to treat Bronchopulmonary Dysplasia (BPD) in premature infants (22) which showed MSC treatment was safe and feasible in a preterm cohort and demonstrated a significant reduction in cytokine levels and lower severity of BPD. This poises MSCs as a strong candidate for use in a clinical trial for treating paediatric liver diseases such as BA, following a display of efficacy in this study (23–25).

#### 3.3.1. MSC Therapy for Biliary Atresia

Biliary atresia (BA) is a progressive fibrosing obstructive cholangiopathy, involving the extrahepatic and intrahepatic biliary systems to varying degrees [80]. Clinical presentation tends to be in the neonatal period and is characterised by direct or conjugated hyperbilirubinaemia, acholic stools, dark urine, variable levels of hepatosplenomegaly and progressive liver failure. Infants who are untreated, rapidly develop progressive fibrosis, leading to portal hypertension and end-stage liver disease, invariably resulting in death within the first two years of life. It has an overall incidence of 1 in 15,000 to 1 in 20,000 in Europe and North America [81].

The aetiology of BA remains incompletely understood but it is likely that there are a number of different mechanisms and factors that contribute to the final common pathway that is identified as the BA phenotype. At King’s College Hospital, we have described a number of distinct variants including a syndromic form—biliary atresia splenic malformation (BASM), cystic BA and more recently, CMV IgM +ve associated BA [82]. The majority of cases, however, do not have any other defining characteristics and are referred to as isolated BA. Environmental and genetic susceptibility factors are thought to interact and orchestrate disease pathogenesis. The key factors that are involved are infectious and immunologic processes [83].

Pathologically, the histologic evidence of lymphomononuclear inflammatory cells infiltrating the vicinity of injured interlobular bile ducts and within the duct epithelium suggests, that in BA, immunologic mechanisms play an important role in bile duct damage [84]. Newly expressed or altered antigens on the surface of bile duct epithelium emerge after an initial viral or toxic insult to the biliary epithelium. This is then presented by macrophages to naïve T lymphocytes. Primed Th1 lymphocytes then organise an immune response through the release of proinflammatory cytokines (IL-2, IFN-γ, TNF-α and IL-12) and recruiting cytotoxic T-cells [85], ultimately causing bile duct epithelial injury. This eventually results in scarring and obliteration of the bile ducts seen in BA.

Early diagnosis is pivotal for the best outcomes from the surgical intervention that is known as Kasai portoenterostomy (KPE). The surgery involves the removal of the atretic extrahepatic biliary tree in an attempt to re-establish bile flow to the intestine by creating a Roux en-Y intestinal conduit. The outcome following this surgery is highly variable in the literature as well as in real world experience, with effective drainage achieved in just over 50% of children [86].

Unfortunately, even with KPE, significant progression of fibrosis and the development of cirrhosis occurs in the majority of patients, requiring liver transplant. BA remains the leading indication for liver transplantation (LT) in children, and at present, no other successful medical treatments have been identified. Despite the major drive to ameliorate the fibro-inflammatory progression in BA, to ultimately reduce the need for LT, no efficacious immunomodulatory treatment for BA is currently available.

Adjuvant medical therapies are a controversial area with arguably marginal evidence to support its use, with institutions around the world adopting individual protocols that are based on experience. As inflammation is thought to be a key factor in the pathogenesis of BA, corticosteroids have been trialled in multiple studies. A randomised, double blind, placebo-controlled trial using low-dose corticosteroids that was conducted in the UK with 71 infants showed no difference in the clearance of jaundice or improvement in the native liver survival/reduction in need for transplantation [87]. Intravenous immunoglobuline (IVIg) administration was trialled as an adjuvant therapy in BA patients in North America as a multicentre Phase Ib/II clinical trial, however failed to show any beneficial effect. As such, it is not routinely used in BA patients post-KPE [88]. None of the treatments above have shown significant improvements in terms of clearance of jaundice, native liver survival or reduction in liver transplantation rates. As such, a safe and efficacious treatment modality is pertinent in this group of patients to improve clinical outcomes.

MSCs as we discussed, preferentially home to damaged tissue, where they serve as a reservoir of growth factors and regenerative molecules. They initiate immunomodulation by targeting a range of innate and adaptive immune cells by direct interaction and by secreting soluble factors [89]. Furthermore, MSCs exhibit anti-fibrotic properties and can directly and indirectly inhibit hepatic stellate cell activation in biliary atresia which contributes to the ongoing biliary atresia. The low immunogenicity that MSCs possess enables allogeneic cells to be used as “off-the-shelf” products. Their safety for use as advanced therapy medicinal products (ATMP) has been widely evaluated.

Lei et al. investigated the anti-fibrotic potential of bone marrow-derived MSCs on liver fibrosis induced by BA [90]. An inflammatory cholangiopathy is induced in the murine model of BA using RRV infection in the newborn BALB/c mice. The pathological phenotype is similar to that in human BA. They demonstrated that BM-MSCs treatment significantly restored liver enzymatic function and bilirubin metabolism and inhibited oxidative stress and alleviated liver fibrosis of the RRV-induced murine model of BA [90]. Collagen IV and COL1A1 were also highly expressed in the liver tissue of the murine model group but BM-MSC treatment significantly reduced their expression. α-SMA expression was also elevated in the BA group but clearly reduced in the BM-MSC treatment group using immunohistochemical staining and Western blotting of liver samples. Pro- inflammatory factors such as TNF-α and TGF-β1 were also significantly inhibited in the BM-MSC group.

Currently there is one Phase I/II clinical trial registered investigating the use of UC-MSC transplantation in patients with BA. Two doses of 1 million MSCs per kg body weight that will be administered at an interval of 6 months.

#### 3.3.2. MSC Therapy for Acute Liver Failure

In acute liver failure, there is rapid loss of function with tissue necrosis and treatment is focused on restoring liver function and preventing disease progression. MSCs have the potential to provide restoration of liver function [91]. The use of MSCs in ALF has been studied in pre-clinical models of mice, rats and monkeys. In a murine model of ALF secondary to acetaminophen poisoning, human UC-MSCs were transplanted intravenously resulting in significantly improved survival rates and alleviated hepatic injury [92]. In a rat ALF model, MSCs were shown to prevent the release of liver injury biomarkers, with recovery of the liver structure [93]. The transplantation of MSCs that were co-cultured with hepatocytes also provided better restoration of liver function, which results in lower levels of aspartate aminotransferase, total bilirubin and alanine aminotransferase (ALT). In another pre-clinical ALF non-human primate model, Guo et al. showed that the early infusion of UC-MSCs could significantly improve hepatic histology, systemic homeostasis and better survival [94]. IL-6 seems to play an important role in initiating and accelerating ALF, whilst UC-MSCs seem to disrupt this inflammatory cascade by inhibiting monocyte activation. Based on these studies, MSCs may have a significant beneficial impact in ALF or as a bridge to transplantation.

The potential of MSCs as a valid treatment modality in replacing hepatocytes in injured liver and effectively rescuing experimental liver failure and contributing to liver regeneration has been demonstrated by many studies (Table 3) and ongoing clinical trials (15 trials currently registered). More research is needed in the realms of sourcing of cells, dosages and the routes of administration to improve the outcomes of this treatment.

#### 3.3.3. MSC Therapy for Cirrhosis

At the crux of liver fibrosis is the transdifferentiation of quiescent HSCs to myofibroblastic HSCs. Regulating HSC activation is a potential therapeutic target for liver fibrosis. As previously discussed, MSCs have the potential to inhibit HSC activation through suppression of their proliferation and stimulating apoptosis by increasing pro-apoptotic proteins. MSCs can also replace dying hepatocytes by differentiating into hepatocyte-like cells. MSCs also exert their anti-inflammatory properties by suppressing the synthesis of pro-inflammatory cytokines such as TNFα, IFNγ and IL-17 and promoting the production of anti-inflammatory cytokines such as IL-4 and IL-10.

Most clinical trials (Table 3) have shown that MSC-based therapies seem to have a beneficial effect on liver fibrosis. In adult studies of alcoholic liver cirrhosis, BM—MSCs significantly improved liver function and reduced the accumulation of collagen [95,96]. In liver cirrhosis secondary to HBV, MSCs were shown to alleviate the expression of fibrotic markers and significantly reduce MELD scores at 48 weeks, whilst also expressing immunomodulatory properties [97]. Whilst little to no side effects were reported in these clinical trials, two studies suggested no significant effect of MSCs in patients with liver cirrhosis [98,99].

Therefore, it remains necessary to conduct, larger-scale clinical trials (50 trials currently registered) covering various conditions leading to cirrhosis, using a range of doses and frequencies, and routes of administration to establish the effectiveness of MSCs in the treatment of liver cirrhosis.

**Table 3:** Mesenchymal stromal cell transplantation: clinical studies in patients with liver disease.

Reference	Liver Disease	Type of Clinical Trial	No of Patients Treated	No of Control Patients	Source of MSCs	Route	Dose	Outcome	Adverse Events
Liver Cirrhosis									
Mohamadnejad et al., 2007 [100]	Decompensated liver cirrhosis	I	4	0	Autologous, bone marrow	Intravenous Peripheral vein	31.73 × 10^6^	Improvement in MELD score *n* = 2, QoL improved in *n* = 4	No side effects
Kharaziha et al., 2009 [101]	End stage liver disease	I–II	8	0	Autologous iliac crest	Intravenous Peripheral vein or portal vein	3–5 × 10^7^, twice	Improvement in MELD score	No side effects
Zhang et al., 2012 [97]	Decompensated liver cirrhosis	Paired controlled study	30	15	UC-MSC	Intravenous, peripheral vein	0.5 × 10^6^ cells/kg body	Reduction in ascites, liver function and MELD score	No side effects
El-Ansary et al., 2012 [102]	Liver cirrhosis secondary to hepatitis C	Phase II RCT	15	10	Bone marrow, autologous	Intravenous	1 × 10^6^ cells/kg body	Improvement in liver function, MELD score	No side effects
Mohamadnejad et al., 2013 [98]	Decompensated cirrhosis	RCT	15	12	Bone marrow, autologous	Intravenous, peripheral vein	1.95 × 10^7^ cells	No significant difference	No side effects
Amin et al., 2013 [103]	Liver cirrhosis secondary to hepatitis C	No control group	20	0	Bone marrow, autologous	Intrasplenic	10 × 10^6^	Significant improvement in liver function tests	No side effects
Jang et al., 2013 [95]	Alcoholic cirrhosis	Phase II clinical trial	11	0	Autologous, bone marrow	Peripheral vein	NA	Improvement in histological appearance, Child score and decrease of TGFb1,a-SMA	No side effects
Wang et al., 2013 [104]	Primary biliary cirrhosis	Single arm trial	7	0	UC-MSCs	Intravenous, peripheral vein	0.5 × 10^6^ cells/kg, three times	Improvement in fatigue and pruritus, and decrease in alkaline phosphatase and GGT	No side effects
Salama et al., 2014 [105]	End-stage liver disease Hepatitis C	RCT	20	20	Autologous, bone marrow	Peripheral vein	NA	Improvement in synthetic function and liver function etsts	No side effects
Xu et al., 2014 [106]	Liver cirrhosis Hepatitis B	RCT	20	19	Autologous, bone marrow	Hepatic artery	NA	Improvement in liver function, increased Treg/Th17 ratio	No side effects
Kantarcioglu et al., 2015 [99]	Liver cirrhosis	No control group	12	0	Autologous, bone marrow	Peripheral vein, intravenous	1 × 10^6^ cells/kg	Improvement in MELD score *n* = 8, no change in liver regeneration or fibrosis at 6m	No side effects
Suk et al., 2016 [96]	Alcoholic liver cirrhosis	Phase 2 RCT	37	18	Autologous, bone marrow	Hepatic artery	5 × 10^7^ once vs twice	Improvement in fibrosis quantification and Child score	No side effects
Sakai et al., 2016 [107]	Liver cirrhosis	Phase I	4	0	Adipose tissue MSC	Hepatic artery	6.6 × 10^5^ cells/kg	Improvement in liver function, HGF and IL6 increased	No side effects
Liang et al., 2017 [108]	Liver cirrhosis secondary to autoimmune disease	No control	26	0	UC-MSC, cord blood MSC, bone marrow MSC	Peripheral intravenous	1 × 10^6^/kg	Improvement in liver function and MELD score	No side effects
Fang et al., 2018 [109]	Decompensated liver cirrhosis, hepatitis B	RCT	50	53	UC-MSCs	Peripheral intravenous	(4.0–4.5) × 10^8^	Improvement in Child score, MELD and liver function	No sie effects
Liver Failure									
Peng et al., 2011 [110]	Hepatitis B liver failure	RCT	53	105	Autologous, bone marrow	Hepatic Artery	NA	Good short term efficacy, no significant long term improvement	No side effects
Amer et al., 2011 [111]	Hepatitis C liver failure	RCT	20	20	Autologous, bone marrow	Intrasplenic, intrahepatic	2 × 10^7^ hepatic lineage committed cells	Signiifcant improvement in ascites, albumin, Child score, MELD score	No side effects
Shi et al., 2012 [112]	Acute on chronic liver failure	Parallel controlled trial	24	19	UC-MSCs	Intravenous, peripheral vein	0.5 × 10^6^ UC-MSCs per kilogram, three times	Significant increase in survival rates, improvement in MELD score	No side effects
Lin et al., 2017 [113]	Acute on chronic liver failure, Hepatitis B	RCT	54	56	Allogenic, bone marrow	Peripheral, intravenous	1.0 to 10 × 10^5^ cells/kg four times	Improved 24 week survival, liver function and decrease in severe infections	No side effects
Liver Transplantation									
Shi et al., 2017 [114]	Allograft liver rejection	RCT	14	13	UC-MSCs	Peripheral intravenous	1 × 10^6^/kg body weight (once or multiple times in *n* = 1)	Improvement in liver function, histology and increased Treg/Th17 ratio	No side effects
Detry et al., 2017 [115]	Liver transplantation	Phase I-II	10	19	Allogenic, bone marrow	Peripheral intravenous	1.5–3 × 10^6^/kg	No improvement in tolerance	No side effects
Zhang et al., 2016 [116]	Ischaemic biliary lesion after liver transplanatation	Phase 1 RCT	12	70	UC-MSC	Intravenous peripheral	1.0 × 10^6^ MSCs per kilogram	Improvement in liver function test and improvement survival	No side effects

### 3.4. Future Directions of MSC Therapy in Liver Disease

As described, multiple clinical trials have verified the safety of MSCs in liver disease with therapeutic efficacy. However, the main issue remains the homing ability of MSCs for which in-depth studies are underway to identify relevant mechanisms of MSC homing and to explore new strategies to improve this.

The key areas of focus include: (1) transplantation route: intravenous infusion of MSCs is easy, economical and can be performed on numerous occasions, but the proportion of cell colonization via this route is low. Portal vein injection may be suitable in patients without risk of portal hypertension, whilst non-systematic homing can be done through intrasplenic puncture injection, the amount is limited each time, and there is an increased risk of bleeding. Hepatic artery injection is also used but is not ideal for multiple treatments. There are still limitations in terms of routes of MSC transplantation and a more individualized case-by-case approach may be appropriate. (2) Optimization of MSC culture conditions: pre-treatment of MSCs have the potential to improve therapeutic efficacy of MSCs, but can affect the phenotypic and paracrine functions, therefore further research is required before clinical use. (3) Modifications of MSCs: this is an active area of research through gene editing and chemical modifications. These methods however may cause biosafety issues and preclinical studies are still needed to safely use these cells [117].

Whilst the safety has been established, the clinical application of MSCs in are still fragmented in terms of the cell source, dose, route, optimal time of inclusion and curative effect. MSCs in clinical use are typically cryopreserved and compared to freshly isolated MSCs, have poorer homing ability and shortens their durability, survival in vivo and tissue repair [118]. Further studies are still needed to investigate the homing mechanism of MSCs and various strategies to improve this, particularly in the pediatric population.

## 4. Conclusions: Future of Cell Therapy

In this review, we have explored the recent advances in studies using hepatocyte and MSC therapies. The use of these modalities requires further optimization from bench to bedside. Important existing issues including preparative measures, optimal timing of injections, longevity of transplanted cells and improved engraftment are key.

The future probably lies in cell-free transplantation. We believe that co-culture techniques like hepatocytes and MSCs could be replaced by small molecules or exosomes that mediate the hepatotrophic, anti-apoptotic and immunomodulatory effects of MSCs. MSCs are known to secrete a variety of factors such as cytokines/chemokines, free nucleic acids, extracellular vesicles and lipids in response to physiological or pathological stimuli. These derived secretomes and extracellular vesicles have similar therapeutic function to MSC-based therapies. Similarly, proregenerative molecules could replace hepatocyte transplantation for acute liver failure. For single gene defects gene therapy will be the future.

## Data Availability

Not applicable.
